# 

**Published:** 1878-05

**Authors:** 


CONTENTS:

Article I.—Plastic Filling, and the Basal Principles of the New Departure. (Continued.) By J. Foster Flagg, D. D. S. .......... 1

Article II.—Some Observations and Experiments Connected with Oral Electricity. By Henry S. Chase, D. D. S., M. D. .......... 18

Article III.—Address before the Alumni Association of the Baltimore College of Dental Surgery. By S. J. Cockerille, D. D. S. .......... 23

Article IV.—Is the Dental Profession Crowded? By Prof. Hodgkin .......... 28

Article V.—Professional Hobbyism. By Henry E. Beach, D. D. S. .......... 31

Article VI.—Substance of Lectures on Gold. (Continued.) By Prof. Hodgkin .... 35

Article VII.—Inquiries Concerning Anæsthetics .......... 39

EDITORIAL, ETC.

Specialism .......... 42
Write It .......... 44
Southern Light .......... 44

BIBLIOGRAPHICAL.

House Air—The Cause and Promotor of Disease .......... 45

MONTHLY SUMMARY.

Carbonic Acid Gas Baths .......... 45
Relations to Life of Aerostatic Pressure .......... 46
Development of Tænia Solium in Man .......... 47
Pierce's Golden Medical Discovery .......... 47
Cerebral Localization .......... 47
The Safety Pocket .......... 47
Lead Poisoning from a Curious Source .......... 48
Relation of Brain Weight to Mental Ability .......... 48

